# Novel allele derived from a Japanese wheat cultivar ‘Asakazekomugi’ for Fusarium head blight resistance without yield penalty and thousand grain weight reduction

**DOI:** 10.1270/jsbbs.24091

**Published:** 2026-02-04

**Authors:** Shizen Ohnishi, Kentaro Horikawa, Naoya Yamaguchi, Chihiro Souma, Yumi Sato, Koichi Morita, Tatsuya Sonoda

**Affiliations:** 1 Hokkaido Research Organization Kitami Agricultural Experiment Station, Kunneppu, Tokoro-gun, Hokkaido 099-1496, Japan; 2 Hokkaido Research Organization Central Agricultural Experiment Station, Naganuma, Yubari-gun, Hokkaido 069-1395, Japan

**Keywords:** Fusarium head blight (FHB), wheat breeding, DNA marker, thousand grain weight (TGW), quantitative trait loci (QTL)

## Abstract

Fusarium head blight (FHB) is a severe disease that affects wheat production. ‘Sumai 3’ has been used as the FHB resistance genetic resource, and DNA markers linked to known quantitative trait loci (QTL) located on 3BS, 6BS, 5AS, or 2DL have been used to detect the ‘Sumai 3’-derived allele. We first developed a near-isogenic line (NIL) of a Japanese cultivar, ‘Kitahonami’, with ‘Sumai 3’ FHB resistant allele at the 2DL-located QTL by recurrent backcrossing. Although the ‘Sumai 3’ allele improved resistance to FHB, it reduced yield and thousand-grain weight (TGW) to unacceptable levels. During the genotype of FHB-resistant QTL in FHB-resistant breeding lines K-1932 and K-1976, we found both lines have a novel allele at the 2DL-located QTL introduced from ‘Asakaze’. We confirmed the co-segregation between the FHB resistance phenotype and the novel ‘Asakaze’-derived allele in the three breeding populations. Furthermore, we developed two sets of NILs, with and without the ‘Asakaze’-derived allele, and examined the FHB resistance, yield, and TGW. The results show that the ‘Asakaze’-derived allele improved FHB resistance similar to the ‘Sumai 3’-derived allele. Surprisingly, the ‘Asakaze’-derived allele did not negatively affect yield and TGW, contrary to the ‘Sumai 3’-derived allele. Wheat breeders can improve FHB resistance without reducing yield and TGW using the ‘Asakaze’-derived QTL allele.

## Introduction

Fusarium head blight (FHB), caused by fungi of the genus *Fusarium* (such as *F. graminearum* and *F. culmorum*, Bockus 2010, Mesterhazy 2024) is one of the most severe diseases of wheat. FHB causes significant economic losses in wheat production owing to reduced yield through floret damage and/or the production of mycotoxins that are harmful to humans. Due to crop rotation and global climate change, FHB epidemics are becoming more severe in major wheat production areas, including North America, South America, and Asia (Bockus 2010, Zhu *et al.* 2019). Because the yield reduction caused by FHB can exceed 30% (Bockus 2010), wheat breeders have attempted to improve FHB resistance on a global scale (e.g., in Europe, the United States, Canada, Brazil, China, and Japan).

Genetic resources for FHB resistance have been identified in China, Europe, Brazil, and Japan (Ban and Suenaga 2000, McCartney *et al.* 2004, Nishio *et al.* 2004, Snijders 1990, Zhu *et al.* 2019). In China, before the 1950s, resistant or moderately resistant landraces ‘Wanshuibai’ (also known as ‘Wanshubai’), ‘Chongyanghongmai’, ‘Pinghujianzimai’, ‘Fanshanxiaomai’, and ‘Taiwanxiaomai’ have been developed (Zhu *et al.* 2019). Although ‘Funo’, an Italian cultivar grown during the 1960s–70s, is moderately susceptible to FHB, its progeny contributes to improved FHB resistance (Zhu *et al.* 2019). ‘Sumai 3’ is widely utilized as an FHB-resistant parent in China, Japan, Canada, the United States, and international breeding programs (Ban 2002, Nishio *et al.* 2004, Yang *et al.* 2006, Zhu *et al.* 2019). ‘Sumai 3’ was developed in 1970 by the Suzhou Institute of Agricultural Sciences in China by crossing the Italian cultivar ‘Funo’ and the Chinese landrace ‘Taiwanxiaomai’. The FHB-resistant Brazilian cultivar ‘Frontana’ (a progeny of the Italian cultivar ‘Mentana’) has been used in wheat breeding programs in the United States to improve resistance to leaf rust. In Japan, where high humidity persists from anthesis to harvest, the FHB-resistant or moderately resistant landraces ‘Nobeokabouzu-komugi’ (also known as ‘Nobeokabozu-komugi’), ‘Nyubai’, and ‘Shinchunaga’ have been known (Ban 2002, Nishio *et al.* 2004, Snijders 1990). In modern breeding programs in Japan, the FHB-resistant breeding line ‘Saikai-165’ is widely used as an FHB-resistant parent (Ban 2002, Ban and Suenaga 2000, Nishio *et al.* 2004). ‘Saikai-165’ was derived from the crossing between ‘Sumai 3’ and ‘Asakazekomugi’ (referred to as ‘Asakaze’) and is believed to have inherited FHB resistance from both parents (Ban and Suenaga 2000).

Although a single gene conferring complete FHB resistance has not yet been identified, several moderately effective quantitative trait loci (QTLs) have been reported. The most well-known QTL, *Fhb1*, found in ‘Sumai 3’, has been cloned and identified as a pore-forming toxin-like (*PFT*) gene (Buerstmayr *et al.* 2003, Rawat *et al.* 2016). Furthermore, using a ‘Sumai 3’-derived line as the resistant parent, *Fhb2* was mapped to chromosome 6BS (Cuthbert *et al.* 2007). Xue *et al.* (2010) and Xue *et al.* (2011) found *Fhb4* and *Fhb5* on chromosomes 4BL and 5AS, respectively, by using ‘Wangshuibai’ as the resistant parent.

Additionally, a 2DL-located QTL has been reported using ‘Wuhan 1’ (Hu *et al.* 2019, Somers *et al.* 2003), ‘CJ9306’ (Jiang *et al.* 2007a, 2007b), ‘Sumai 3’ (Suzuki *et al.* 2012), ‘Soru#1’ (He *et al.* 2016), ‘Yangmai 158’, and ‘Yangmai 12’ (Hu *et al.* 2023, Yan *et al.* 2021) as the FHB-resistant parents. In these studies, the QTL was located near *gwm539*, with the exception of Somers *et al.* (2003). He *et al.* (2014) reported that a 2DL-located QTL may contribute to FHB resistance in breeding programs in China. McCartney *et al.* (2004) reported that multiple alleles exist on *gwm539*, which is the closest marker to the resistance 2D-located QTL. For example, the old Japanese cultivar ‘Asakaze’ represents a 2 bp longer PCR product than ‘Sumai 3’ by *gwm539* (McCartney *et al.* 2004). However, most of these multiple alleles were not identified as resistant or susceptible, and breeders would treat them as susceptible alleles.

Although the 2DL-located QTL confers resistance, it simultaneously reduces the thousand-grain weight (TGW), as reported by Suzuki *et al.* (2012) in analyses of the ‘Sumai 3’-derived allele at the 2DL-located QTL. Hu *et al.* (2023) reported that the FHB resistance allele at the 2DL-located QTL, derived from the Chinese cultivar YM12, also reduced TGW. Thus, it is challenging to simultaneously obtain sufficient TGW and FHB resistance using the 2DL-located QTL.

In northern Japan, the wheat breeding program of the Hokkaido Research Organization (HRO) has been aimed at improving FHB resistance since 1989, and has bred FHB-resistant breeding lines. Although these FHB-resistant lines tended to show a long-term reduction in TGW, the elite winter wheat lines K-1932 and K-1976 exhibited acceptable TGW and high FHB resistance. In this study, we investigated K-1932 and K-1976 at the 2DL-located FHB resistance QTL. We found a novel resistance allele originating from Japanese landraces that was not associated with TGW reduction or yield penalty.

## Materials and Methods

### Plant materials

‘Kitahonami’, a Japanese wheat cultivar, was used as a recurrent parent to produce the near-isogenic line (NIL) having 2DL-located QTL of ‘Sumai 3’. ‘Kitahonami’ is a soft red winter wheat cultivar for Japanese noodles in Hokkaido. ‘Kitahonami’ has been released in 2006 (Yanagisawa *et al.* 2007) and is currently a leading variety in Hokkaido. ‘Sumai 3’ is an FHB-resistant cultivar and was introduced from the National Agricultural Research Center for the Kyushu Okinawa Region to the HRO. ‘Sumai 3’ was used as a donor of the FHB resistance allele.

The elite FHB-resistant breeding lines K-1932 and K-1976, bred in the HRO breeding program, were used to genotype FHB-resistant QTLs. K-1932 and K-1976 were derived from crosses of Kitami-82/18028 and Kitami-89/22157, respectively. These two lines were used for the genotype analysis of FHB-resistant QTL alleles located on chromosomes 2DL, 3BS, and 5AS.

Three breeding populations, KC-6153 (K-1976/K-1993), KC-6055 (Kitami-93/K-1961//K-1976), and KC-6070 (K-1962/K-1929//K-1976), were used to examine the relationships between the ‘Asakaze’-derived 2DL-QTL allele and the phenotype of FHB resistance. KC-6070 was also used to evaluate the relationship between the ‘Asakaze’-derived 2DL-QTL allele and TGW. The parental line K-1976 is FHB-resistant (as described above), and K-1993, Kitami-93, K-1961, K-1962, and K-1929 are FHB-susceptible elite breeding lines generated in the HRO.

The breeding population KC-5997 was used to generate two sets of NILs (W22308-c and W22308-b, and W22309-c and W22309-b) to evaluate the relationships between the genotype of 2DL-located QTL and FHB resistance, yield, and TGW phenotypes. The population KC-5997 was obtained by crossing K-1976 with Kitami-94. K-1976 is the FHB-resistant line above described, and Kitami-94 is an elite breeding line generated by backcrossing Kitahonami as a recurrent parent and Madsen as a Wheat Yellow Mosaic Virus (WYMV)-resistant donor (Suzuki *et al.* 2022). Kitami-94 is FHB-susceptible, similar to Kitahonami, and does not have a resistance allele at any known FHB-resistant QTL.

The ancestral lines of ‘Asakaze’ are listed by Nonaka *et al.* (1979), and germplasm resources for genotyping were obtained from the NARO Genebank, Tsukuba, Japan (Supplemental Table 1). The other working stocks were obtained from the wheat breeding program of the HRO.

### Field management and yield trial

Field experiments were conducted at the Kitami Agricultural Experiment Station (Kitami AES) in Hokkaido, Japan (43.75° N, 143.72° E). This location has warm temperatures, moderate radiation, andosol soil, and rainfed field conditions. The average total rainfall amount during the period from April to July was 255 mm (Sakaino Station Automated Meteorological Data Acquisition System).

Seeds for the yield trials were sown in late September each year, and wheat plants were harvested in August. Weeds and diseases were controlled through herbicide and pesticide application following standard protocols in this area. Starter fertilizer was applied (57 kg/ha N, 175 kg/ha P_2_O_5_, and 70 kg K_2_O), and additional nitrogen was applied in April (50 kg/ha N) and at the pre-boot stage (50 kg/ha N), achieving a maximum yield of nearly 8 t/ha without lodging. The number of replications for the yield trial was two, two, and three for 2014, 2015, and 2016, respectively, for ‘Kitahonami-2DL’ and two for evaluations of residual heterozygote-derived NILs.

### Evaluation of FHB resistance

All evaluations of FHB resistance were conducted at Kitami AES. Seeds were sown in mid-to late September in plots with rows of 50 cm or 1 m in length and placed 60 cm apart. Watering using a sprinkler system began in late May, just before heading. The sprinkler system was placed in a grid of approximately 6 × 6 m at a height of 1.2 m. Watering was performed hourly for 7 min, except during the 3 h period before inoculation by spraying.

The plants were inoculated primarily using the spray method to ensure equal intensity. The oat method was adopted in some experiments to inoculate large numbers of lines. We confirmed that the results of the two methods were highly correlated, and the method adopted for each experiment is described in Supplemental Table 2. The highly pathogenic *Fusarium graminearum* strain TYK101-1 (producing deoxynivalenol) was collected from Hokkaido, Japan. For spray inoculation, spores on the TYK101-1 medium were collected and diluted with water to a final concentration of 1 × 10^4^ spores/L. Immediately before inoculation, 1/5000 volume of Tween-20 was added to the diluted spore solution. At 17:00 on the day when more than 50% of the florets in the plot had bloomed, 100 mL of the diluted spore solution was sprayed into the plot with 1-m-long rows. The severity of FHB was evaluated three weeks after inoculation. Twenty spikes per plot were evaluated and scored as follows: 0, no symptoms; 1, single florets were bleached; 2, two florets were bleached; 3, three–five florets were bleached; 4, 50% of the florets were bleached; 5, 60–70% of the florets were bleached; 6, 70–80% of the florets were bleached; 7, 80–90% of the florets were bleached; and 8, almost all florets were bleached. The FHB severity was calculated as [average score]/8 × 100 (with values ranging from 0 to 100).

For the oat method, a block of the stocked TYK101-1 medium was placed in a 500 mL flask filled with 200 mL of oats after autoclaving twice for 60 min at 121°C with 100 mL of water. After incubation for 2 weeks at 25°C, 5 g of the oat medium was inoculated on the soil surface of the plot with 1-m-long rows in late May. The incidence of FHB was scored by visual observation based on the percentage of bleached florets (0%, 5%, 10%, 20%, 30%, 40%, 50%, 60%, 70%, 80%, 90%, or 100%) in the entire plot. The scoring was conducted on July 7, 2023.

### Genotyping of QTL alleles

The SSR markers linked to the QTLs shown in Table 1 were used to determine their genotypes. The length of PCR product of each DNA marker is listed in Table 1, which differs from the actual amplifying DNA size and varies depending on experimental conditions due to the fluorescent dyes attached to the primers (e.g., ‘Sumai 3’ allele *Xgwm539* was 141 bp under our experimental conditions using FAM as the dye, compared with 144 bp in McCartney *et al.* 2004). As the nearest DNA marker for the 2DL-located QTL, *gwm539* was used to generate Kitahonami-2DL, two sets of NILs (W22308-c and W22308-b or W22309-c and W22309-b), and the three sets of sibling lines described above. The DNA marker *gwm539* was also used to genotype ancestral lines of K-1932, K-1976, and ‘Asakaze’. Three DNA markers, *gwm539*, *gwm493*, and *gwm304* were used to evaluate QTLs on 2DL, 3BS, and 5AS for K-1932 and K-1976. The other DNA markers used in the analyses have been described previously (Suzuki *et al.* 2012).

Primer sequences are listed in Supplemental Table 3. Wheat DNA was extracted from young leaves as described previously (Suzuki *et al.* 2012). PCR was conducted using AmpliTaq Gold 360 DNA polymerase (Applied Biosystems, Foster City, CA, USA) at an annealing temperature of 56°C. A portion of the PCR products was analyzed using an ABI Prism 3500 Genetic Analyzer (Applied Biosystems) and GeneMapper software (Applied Biosystems), as described previously (Suzuki *et al.* 2015). For the genotyping of lines or cultivars, leaf samples were collected from three different individual plants and bulked before DNA extraction. The lines were judged to have segregated if two peaks of PCR products corresponding to the parental lines were detected.

### Statistical analyses

Analysis of variance (ANOVA) and *t*-test implemented in JMP 11.0 (SAS Institute, Cary, NC, USA) and Excel 2019 (Microsoft, Redmond, WA, USA) were used for the statistical analyses. The ANOVA design and results are presented in Supplemental Table 4.

## Results

### Effect of the ‘Sumai 3’ allele at 2DL-QTL on FHB resistance and agronomic traits

**Fig. 1.**
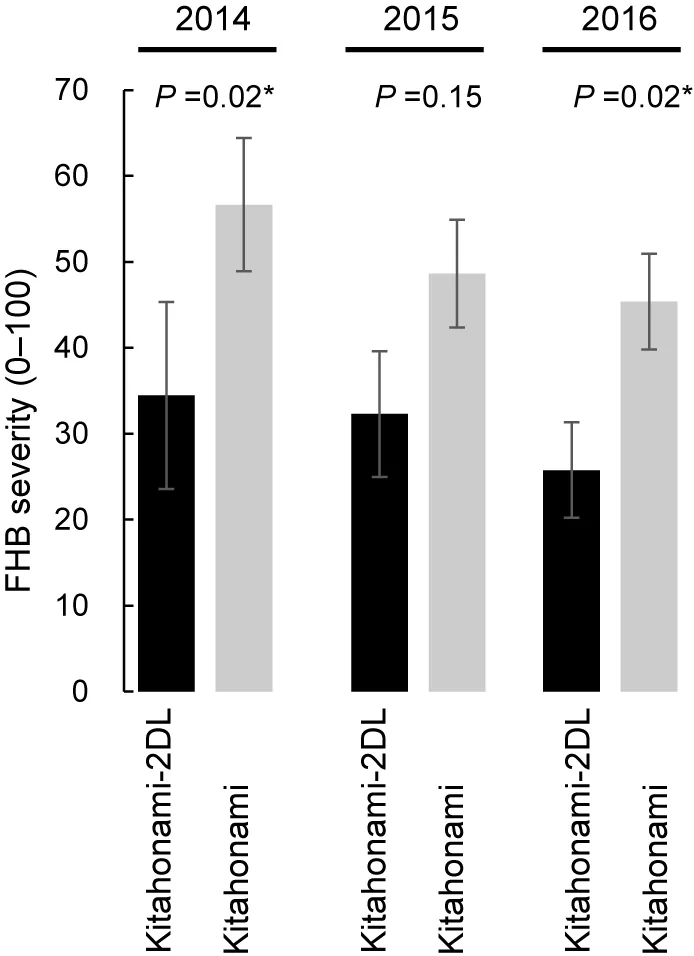
Differences in Fusarium head blight (FHB) severity between NILs with the 2DL-located QTL for FHB resistance. ‘Kitahonami-2DL’ is a line bred by backcrossing with ‘Kitahonami’ as the recurrent parent and ‘Sumai 3’ as the FHB-resistant allele donor. Sowing year and *P*-values of the *t*-test between evaluated lines for each year are shown above the bars. The asterisk indicates significant differences (*P* < 0.05). Error bars indicate standard error of means.

We introduced a ‘Sumai 3’-derived allele at the 2DL-located QTL into ‘Kitahonami’ by backcrossing and then generated a NIL ‘Kitahonami-2DL’. At each backcross generation, DNA markers were used for detecting heterozygous BC_n_F_1_ plants harboring the ‘Sumai 3’ allele at all QTLs on each chromosome (*gwm539* for 2DL, *gwm493* for 3BS, *gwm304* for 5AS) (Table 1). The BC_5_F_3_ population was generated by the single-seed descent method using BC_5_F_1_ plants, and 334 single plant-derived lines were generated using BC_5_F_4_. Among 334 lines, a single homozygous line was selected as ‘Kitahonami-2DL’, harboring the ‘Sumai 3’ allele (resistant allele) at the 2DL-located QTL and the ‘Kitahonami’ allele (susceptible allele) at the other QTLs (3BS and 5AS). Fig. 1 shows the differences in FHB severity between ‘Kitahonami’ and ‘Kitahonami-2DL’. The ‘Kitahonami-2DL’ exhibited significantly lower FHB severity (increased resistance) than that of ‘Kitahonami’ at the FHB evaluation field for three years. This indicates that the 2Dl-located QTL from ‘Sumai 3’ is stably effective to improve FHB resistance. We also evaluated agronomic traits for yield performance in an FHB-controlled field (Table 2). For the years yield trials, the days to heading, days to maturity, and plant height were not significantly different between the two lines. However, significantly, ‘Kitaonami-2DL’ exhibited a 5.1 g lower TGW on three years average than those of ‘Kitahonami’ (Table 2). We also found that the introduction of ‘Sumai 3’-derived allele reduced yield by 8% on average, in addition to TGW (Table 2). A reduction in yield was observed over all three tested years (Table 2). Since the number of grains per square meter was almost the same between the two lines (Table 2), the decline in TGW seems to be responsible for the reduction in yield.

### Genotype of known QTLs of the elite FHB-resistant lines

K-1932 and K-1976 were elite FHB-resistant breeding lines without a reduction in TGW. Both lines have an origin from the FHB-resistant line ‘Saikai-165’, through a crossing between ‘Sumai 3’ and ‘Asakaze’, known as an FHB-resistant cultivar, in the Kyushu region in Japan. Since any DNA marker selection with known FHB-resistant QTLs had not applicated to K-1932 and K-1976, we determined the genotypes of FHB-resistant QTLs located at 2DL, 3BS and 5AS (Table 1) in K-1932 and K-1976. Table 3 shows the results of the genotyped FHB-resistant QTLs. K-1932 and K-1976 harbored the ‘Sumai 3’-derived allele on the 3BS- or 5AS-located QTL, respectively (Table 3). However, both lines did not have the ‘Sumai 3’-derived allele at the 2DL-located QTL. Instead, both lines shared the allele identical to that of ‘Asakaze’ (Table 3, Supplemental Fig. 1).

**Fig. 2.**
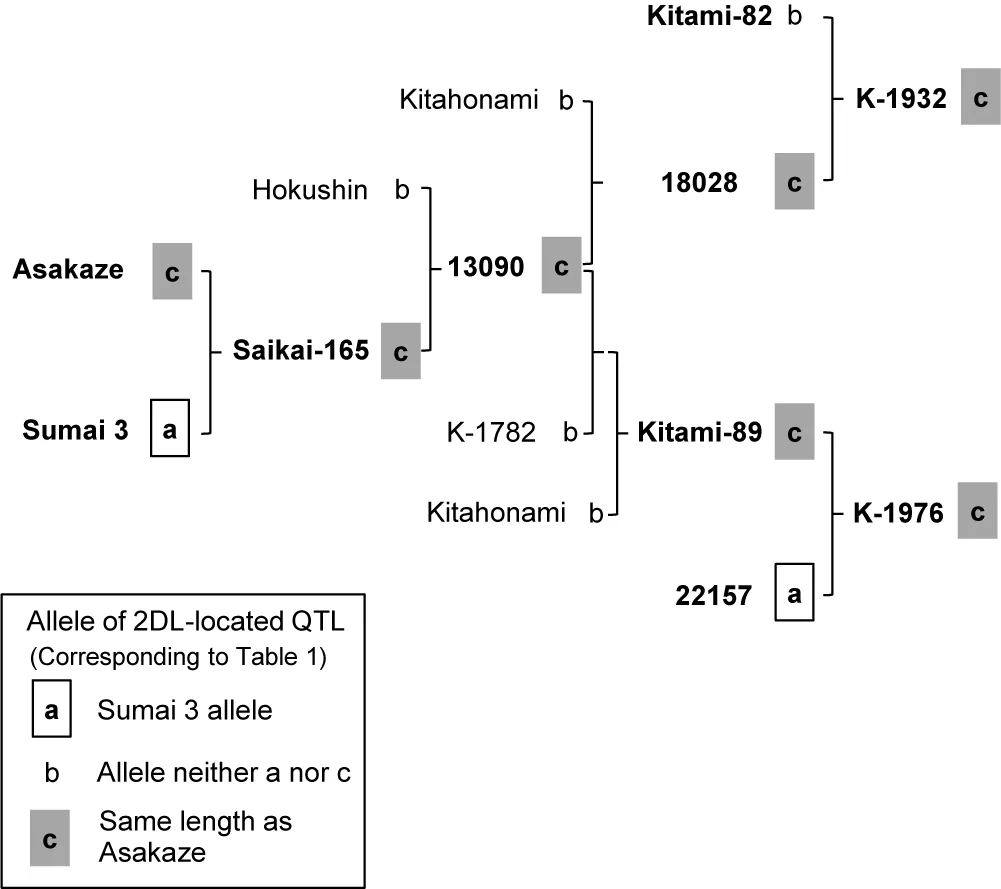
Pedigree of K-1932 and K-1976 with the 2DL-located QTL genotype (*gwm539*). The genotype is denoted as a, ‘Sumai 3’ allele; b, allele neither a nor c; and c, same length as ‘Asakaze’ (Table 1). Pedigrees were drawn using the breeding data set of HRO. Abbreviated cultivar names: ‘Asakaze’, ‘Asakazekomugi’. Lines defined as moderately to highly resistant to FHB based on evaluations by the HRO breeding program, Nishio *et al.* (2004), or Ban (2002) are indicated in bold font.

To determine the origin of this allele, we genotyped the ancestral lines K-1932 and K-1976. The results show that the allele at the 2DL-located QTL in K-1932 and K-1976 was introduced from ‘Asakaze’ via the FHB-resistant breeding lines ‘Saikai-165’ and 13090 (Fig. 2). The fact that two highly FHB-resistance lines have a common allele derived from ‘Asakaze’ instead of that of ‘Sumai 3’ at the 2DL-located QTL indicates the possibility that this ‘Asakaze’-derived allele may contribute to FHB resistance in K-1932 and K-1976.

### Effect of the ‘Asakaze’-derived allele at 2DL-QTL on FHB resistance and agronomic traits

**Fig. 3.**
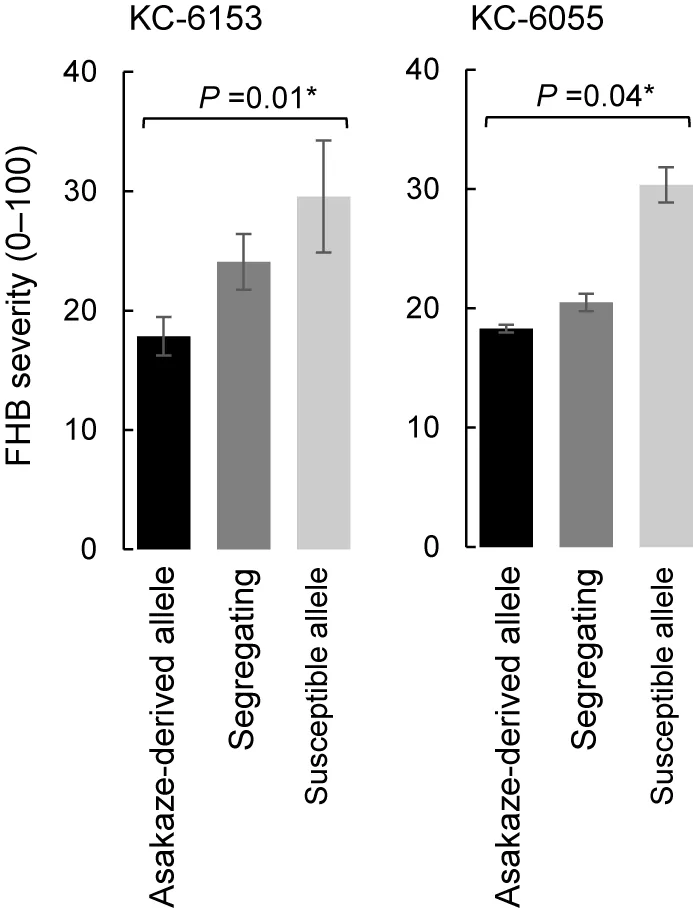
Differences in FHB severity of sibling lines by 2DL-located QTL genotype for FHB resistance. Averages for each genotype (‘Asakaze’-derived allele; susceptible allele; and segregating within a line, Table 1) are shown. The evaluations were conducted in the 2022 sowing season. The names of each cross (KC-6153 and KC-6055) and *P*-values of one-way analysis of variance (ANOVA) are indicated above the bars. The asterisk indicates significant differences (*P* < 0.05). Error bars show standard error of means. Lines with the ‘Asakaze’-derived allele show lower FHB severity than the other lines.

To demonstrate the relationship between the ‘Asakaze’-derived 2DL-QTL allele and FHB-resistant phenotype, we examined the sibling lines derived from two breeding populations, KC-6153 (K-1976/K-1993) and KC-6055 (Kitami-93/K-1961//K-1976). A set of F_6_ 78 lines derived from 78 individual plants of the KC-6153 population and F_7_ 45 lines derived from 45 individual plants of the KC-6055 population were used for FHB resistance evaluation and genotyping of the 2DL-located QTL. Using the *gwm539* marker, for the KC-6153 population, we obtained 44 lines with the ‘Asakaze’-derived allele, nine lines without the ‘Asakaze’-derived allele (susceptible allele), and 25 segregating lines. In the KC-6055 population, 25 lines with the ‘Asakaze’-derived allele, 10 lines without the ‘Asakaze’-derived allele (susceptible allele), and 10 segregating lines were acquired. FHB severity of the two sets of sibling lines according to genotype is shown in Fig. 3. Sibling lines harboring the ‘Asakaze’-derived allele exhibited lower disease severity than lines with susceptible alleles in both populations (Fig. 3). The segregating lines showed intermediate severity between the ‘Asakaze’-derived and susceptible alleles. One-way ANOVA for each population indicated that the ‘Asakaze’-derived allele significantly affected FHB resistance (Fig. 3, Supplemental Table 4). These results demonstrate that the ‘Asakaze’-derived allele at the 2DL-located QTL is resistant allele.

We further confirmed the effects of the ‘Asakaze’-derived allele using another set of sibling lines developed from the population, KC-6070 (K-1962/K-1929//K-1976). Seven lines (W22261, W22262, W22263, W22264, W22265, W22266, and W22267) from the F_6_ generation derived from F_5_ independent individual plants were used (Supplemental Fig. 2). The severity of FHB, TGW, and the genotype of the 2DL-located QTL in each line are shown in Table 4. Three lines, W22261, W22265, and W22266, had the ‘Asakaze’-derived allele (c in Table 1); two lines, W22262 and W22267, had non-‘Asakaze’-derived alleles (susceptible allele b in Table 1); and two lines, W22263 and W22264, exhibited segregation. Lines with the ‘Asakaze’-derived allele showed 30% lower FHB severity on average, and the ANOVA indicated that the genotype of the 2DL-located QTL significantly affected FHB severity (*P* = 0.08, Table 4). These results confirmed that the ‘Asakaze’-derived allele at the 2DL-located QTL is resistant allele. Furthermore, the TGW of lines with ‘Asakaze’-derived alleles was 2.1 g greater than that of the susceptible allele, although there were significant differences in TGW among the three genotypes (Table 4).

Finally, we evaluated two pairs of NILs for the 2DL-located QTL. To create NILs within a short period, we adopted residual heterozygote-derived NILs rather than backcross-derived NILs. Two sets of NILs were produced from the KC-5997 population, generated by crossing K-1976 and Kitami-94. In the F_5_ generation, five lines originated from F_4_ independent individual plants (Supplemental Fig. 3). Among the five lines, segregation of the genotypes of the DNA marker *gwm539* was observed in two lines, W22308 and W22309. Single plant-derived lines were prepared for W22308 and W22309 at F_6_. Among 4 lines for each family, lines with fixation of the ‘c’ allele (derived from the parent K-1976, Table 1) at *Xgwm539* were selected as W22308-c and W22309-c and lines with fixation for ‘b’ (derived from the parent Kitami-94, Table 1) were selected as W22308-b and W22309-b (Supplemental Fig. 3). These two sets of NILs (W22308-c, W22308-b, W22309-c, and W22309-b) were evaluated for FHB resistance (Table 5). The lines with the ‘Asakaze’-derived alleles (W22308-c and W22309-c) showed 13% and 9% lower FHB severity than those with the Kitami-94-derived susceptible alleles (W22308-b and W22309-b), respectively. The ANOVA results revealed a significant effect of the genotype of the 2DL-located QTL on FHB severity (*P* = 0.01, Table 5). This confirmed that the ‘Asakaze’-derived allele at the 2DL-located QTL was resistant allele.

We investigated whether the ‘Asakaze’-derived allele exhibits a negative effect on agronomic traits containing TGW and yield, as observed in the ‘Sumai 3’-derived resistant allele. The lines with the ‘Asakaze’-derived allele showed an average yield of 9.64 t/ha for the two NILs, which was almost the same as the average yield of the lines with susceptible alleles (Table 5). ANOVA showed no effect of genotype at the 2DL-located QTL on the yield (*P* = 0.67). For TGW, the difference between genotypes was only 0.1 g, and no significant effect on TGW was detected by ANOVA (Table 5). The results in Table 5 clearly indicate that the ‘Asakaze’-derived 2DL-located QTL allele did not negatively influence agronomic traits, including days to maturity, plant height, yield, and TGW, except for days to heading, which differed by an average of 1 d (*P* = 0.09) (Table 5).

Based on the present results through two different sowing seasons (an experiment of sibling lines in 2021 and that of NILs in 2023), we conclude that the ‘Asakaze’-derived allele at 2DL-located QTL does not negatively affect TGW, in contrast to the ‘Sumai 3’-derived resistant allele.

### Origin of the ‘Asakaze’-derived allele at 2DL-QTL

**Fig. 4.**
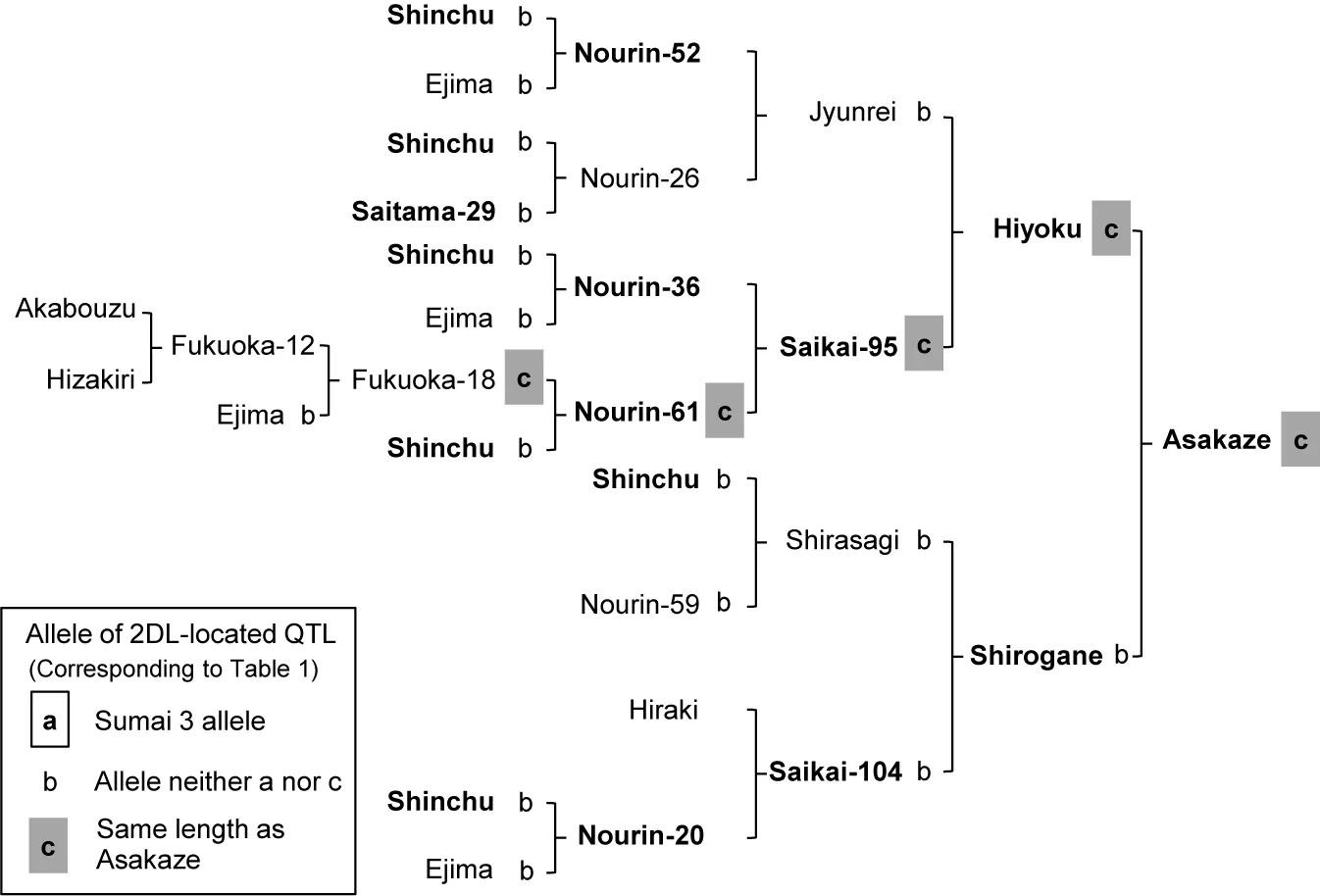
Pedigree of ‘Asakaze’ with the 2DL-located QTL genotype (*gwm539*). The genotype is denoted as a, ‘Sumai 3’ allele; b, allele neither a nor c; and c, same length as ‘Asakaze’ (Table 1). Pedigrees were drawn using the parentage described by Nonaka *et al.* (1979). Abbreviated cultivar names: ‘Asakaze’, ‘Asakazekomugi’; Hiyoku, ‘Hiyokukomugi’; Shirogane, ‘Shiroganekomugi’; Jyunrei, ‘Jyunreikomugi’; Shirasagi, ‘Shirasagikomugi’; Hiraki, ‘Hirakikomugi’; Ejima, ‘Ejimajinriki’; Shinchu ‘Shinchunaga’; Fukuoka-18, ‘Fukuokakomugi-18’; and Fukuoka-12, ‘Fukuokakomugi-12’. Lines defined as moderately to highly resistant to FHB based on evaluations by the HRO breeding program, Nishio *et al.* (2004), or Ban (2002) are indicated in bold font.

To reveal the origin of the ‘Asakaze’-derived allele, we investigated the genotypes of key ancestral germplasm of ‘Asakaze’ using the DNA marker *gwm539*. The c allele was derived from ‘Fukuoka-18’ and passed through ‘Nourin-61’, ‘Saikai-95’, and ‘Hiyokukomugi’ (Fig. 4); it did not originate from other FHB-resistant landraces, including ‘Shinchunaga’ (Fig. 4). Fukuoka-18 is an old Japanese breeding line, and the origin of the c allele was deduced to be the Japanese landrace ‘Akabouzu’ (also referred to as ‘Akabozu’) or ‘Hizakiri’ based on the pedigree of ‘Fukuoka-18’ (Fig. 4).

## Discussion

‘Sumai 3’ is considered an effective germplasm resource for FHB resistance breeding. In this study, we demonstrated that the ‘Sumai 3’-derived allele at 2DL-located QTL reduced TGW (Table 2), consistent with previous findings (Suzuki *et al.* 2012). Furthermore, we first revealed that the ‘Sumai 3’-derived allele showed a negative effect on yield: it reduced yield due to lower TGW. Thus, the ‘Sumai 3’-derived allele should be difficult to apply in practical breeding programs for FHB resistance.

In this study, we identified a novel resistance allele at the 2DL-located FHB resistance QTL, which is distinct from the ‘Sumai 3’-derived allele based on the DNA marker *gwm539* (Fig. 3, Tables 1, 4, 5, Supplemental Fig. 1). Notably, this ‘Asakaze’-derived novel allele exhibited no negative effects on TGW nor yield as seen in the ‘Sumai 3’-derived allele (Tables 4, 5). We also demonstrated that the novel allele was introduced from a Japanese old cultivar ‘Fukuoka-18’ through ‘Nourin-61’ and ‘Saikai-95’ to the elite breeding lines of Hokkaido (Figs. 2, 4).

McCartney *et al.* (2004) investigated the genotype of *gwm539* in 78 key FHB-resistant accessions, including ‘Sumai 3’, ‘Wangshubai’, ‘Fanshanxaomai’, ‘Wuhan 1’, ‘Nubai’, ‘Frontana’, and ‘Funo’. Only ‘Asakaze’ showed an PCR product that was 2 bp longer than that of ‘Sumai 3’. They also reported that ‘Wuhan 1’, a cultivar from which the 2DL-located QTL was first detected (Hu *et al.* 2019, Somers *et al.* 2003), had an PCR product identical with ‘Sumai 3’ with *gwm539*. For the FHB-resistant allele in ‘Wuhan 1’, the precise position of the QTL is reported near *wmc144* to *wmc245*, different from *gwm539* (He *et al.* 2014, Somers *et al.* 2003). However, ‘Asakaze’ possesses an allele different from that of ‘Wuhan 1’ within *wmc144* to *wmc245* (McCartney *et al.* 2004). Thus, the ‘Asakaze’-derived novel resistant allele is completely different from that of ‘Wuhan 1’. Another well-known allele at the 2DL-located QTL is that derived from ‘CJ9306’ (Jiang *et al.* 2007a, 2007b), which is frequently found in Chinese FHB-resistant germplasm (He *et al.* 2014). Although ‘Asakaze’ and ‘CJ9306’ have a common ancestral parent ‘Shinchunaga’, our results indicate that the ‘Asakaze’-derived allele did not originate from ‘Shinchunaga’ (Fig. 4). Moreover, through capillary electrophoresis using the ABI 3500xl Genetic Analyzer with single-base pair resolution, He *et al.* (2014) reported that the *gwm539* genotype of ‘CJ9306’ was identical to that of ‘Sumai 3’ (with an amplified length of *gwm539* of 127 bp for ‘CJ9306’ under their experimental conditions). Our results, together with those of the previous studies, suggest that the ‘Asakaze’-derived allele is a unique novel resistant allele and is distributed only within Japanese wheat germplasm.

In this study, we wish to argue that the ‘Asakaze’-derived 2DL-locaed QTL allele does not adversely affect TGW and yield as seen in the ‘Sumai 3’-derived allele. The evaluation of agricultural traits such as TGW and yield potential requires surveys spanning several years, not just a single year. In this study, we demonstrated through a three-year trial that the ‘Sumai 3’-derived allele negatively impacts yield potential, with the cause being a reduction in TGW. We also demonstrated that the ‘Asakaze’-derived allele does not adversely affect TGW by two-year experiments (Tables 4, 5). However, we investigated only one year for the effect of the ‘Asakaze’-derived allele on the potential yield (Table 5).

Despite this limitation, we deduced that the ‘Asakaze’-derived allele was superior in terms of yield than that of ‘Sumai 3’. Although assessed in only one year, the ‘Asakaze’-derived allele showed a yield comparable to that of lines carrying the susceptible allele (Table 5), whereas lines with the ‘Sumai 3’-derived allele consistently exhibited severe reductions in both yield and TGW across all three tested years (Table 2). Additionally, in the 3-year experiment involving the ‘Sumai 3’-derived allele (Table 2), the yield reductions were due to the decrease in TGW and not to a decline in thousand grains per square meter, when divided into yield components (Table 2). At 2 years (Tables 4, 5), the ‘Asakaze’-derived allele did not affect TGW; accordingly, we deduced that the ‘Asakaze’-derived allele did not affect the yield. Based on these results, we concluded that the ‘Asakaze’-derived allele did not affect TGW or yield. Evaluating the yield potential over multiple years remains challenging.

What is the cause of the difference in the effects of the ‘Asakaze’-derived allele and the ‘Sumai 3’-derived allele on TGW and yield potential? This phenomenon can be explained in several ways. One hypothesis is that the FHB-resistance genes within this QTL are distinct, with each exhibiting different pleiotropic effects on agronomic traits. The second hypothesis is that although the FHB resistance genes are the same, the linked agricultural trait-related genes differ. If the resistance gene for this QTL could be cloned, the answer would naturally be as follows. Furthermore, it is necessary to directly compare the degree of FHB-resistance conferred by the two alleles, ‘Asakaze’-derived and ‘Sumai 3’-derived. We need to develop the NILs with the two alleles sharing the same genetic background.

In this study, we identified the origin of the ‘Asakaze’-derived allele as the old Japanese cultivar ‘Fukuokakomugi-12’, developed in Kyushu in southwestern Japan (Fig. 4). We were also able to deduce that the origin of the ‘Asakaze’-derived allele was the Japanese landrace ‘Akabouzu’ or ‘Hizakiri’ based on a pedigree analysis of ‘Fukuoka-18’ (Fig. 4). Additionally, breeding lines and cultivars harboring the ‘Asakaze’-derived allele (‘Fukuokakomugi-12’, ‘Fukuokakomugi-18’, ‘Nourin-61’, ‘Saikai-95’, ‘Hiyokukomugi’, ‘Asakaze’, and ‘Saikai-165’; Figs. 2, 4) were bred in Kyushu. It is likely to happen that in southwestern Japan, where FHB is endemic, the ‘Asakaze’-derived allele had been introduced from the Japanese landrace to old cultivars and then passed to cultivars and breeding lines developed in the southwestern part of Japan. Whether recent modern cultivars bred in southwest Japan after ‘Asakaze’ also harbor the ‘Asakaze’-derived allele is worthy of further investigation. Zhu *et al.* (2019) reported that instead of the ‘Sumai 3’-derived allele, a resistance allele derived from ‘Shinchunaga’ with *Fhb1* on chromosome 3BS contributed to improved FHB resistance in a Chinese wheat breeding program. ‘Shinchunaga’ is a Japanese landrace originating in southwestern Japan. Southwestern Japan may be a good source of FHB-resistant alleles due to its warm and humid climate. The absence of the ‘Asakaze’-derived allele among Chinese landraces or progenies of Chinese landraces is noteworthy (McCartney *et al.* 2004), given that Japanese wheat is believed to have originated in China (Nakamura 2002).

The ‘Asakaze’-derived allele has not been a focus of research, even though various old cultivars harboring the ‘Asakaze’-derived allele are found in southwestern Japan. The small size difference of only 2 bp between the ‘Asakaze’-derived and ‘Sumai 3’-derived alleles can cause misidentification when using polyacrylamide gel electrophoresis. For example, a previous study using this method has incorrectly reported that the genotype of *gwm539* in ‘Asakaze’ is identical to that of ‘Sumai 3’ (Niwa *et al.* 2018). Another explanation is that the ‘Asakaze’-derived allele has been described as susceptible because it is different from the ‘Sumai 3’-derived allele. Before the present study, the ‘Asakaze’-derived allele at the 2DL-located QTL was regarded as a susceptible allele in the breeding program of HRO.

The ‘Asakaze’-derived allele functions as a resistance allele without any adverse effects on agronomic traits. This is an important discovery for breeding FHB-resistant wheat. The study results can guide breeding programs aimed at improving FHB resistance using the ‘Asakaze’-derived allele.

## Author Contribution Statement

KH designed the first hypothesis and SO designated experiments. KH, CS, and NY evaluated the QTL genotypes. SO, YS, KM, and TS evaluated agronomic traits and FHB resistance. SO analyzed the data and wrote the manuscript.

## Supplementary Material

Supplemental Figures

Supplemental Tables

## Figures and Tables

**Table 1.** Evaluation and descriptions of Fusarium head blight (FHB) resistance QTLs used in this study

QTL location	DNA marker	Descriptions of allele in this study	Length of PCR product (bp)	References
2DL	*gwm539*	**a**: derived from Sumai 3	141	144 bp at (1)
		**b**: neither **a** nor **c** allele (Susceptible allele)	153 or other length	(2) and this paper
		**c**: same length as Asakazekomugi	143	146 bp at (1) and this paper
3BS	*gwm493*	**a**: derived from Sumai 3	210	213 bp at (1)
		**b**: not **a** allele (Susceptible allele)	155	(2) and this paper
5AS	*gwm304*	**a**: derived from Sumai 3,	233 + 235	233 bp at (1)
		**b**: not **a** allele (Susceptible allele)	217 + 219	(2) and this paper

Reference; (1) McCartney *et al.* (2004) and (2) Suzuki *et al.* (2012).

**Table 2.** Effect of the ‘Sumai 3’-derived allele on agricultural traits at the yield trial with NILs

Year	Line	Genotype of 2DL-located QTL	Days to heading	Days to maturity	Plant height (cm)	Yield (t/ha)	Yield percentage [a/b] (%)	TGW (g)	Difference in TGW [a – b] (g)	Thousand grains per square meter
2014	Kitahonami-2DL*^b^*	a	267	307	76.0	4.74	92	33.5	–3.6	14.2
	Kitahonami	b	267	307	77.4	5.13		37.1		13.8
2015	Kitahonami-2DL	a	260	306	76.2	6.61	91	39.0	–6.0	16.9
	Kitahonami	b	260	305	76.9	7.30		45.0		16.2
2016	Kitahonami-2DL	a	261	309	86.9	9.78	93	37.3	–5.7	26.2
	Kitahonami	b	261	311	86.6	10.50		43.0		24.4
Average	Kitahonami-2DL	a	263	307	79.7	7.04	92	36.6	–5.1	19.1
	Kitahonami	b	262	308	80.3	7.64		41.7		18.2
***P*-value*^a^***			**0.67**	**0.32**	**0.60**	**0.07^†^**		<0.01**		**0.28**

*^a^* *P*-value is for the effect of ‘Genotype of 2DL-located QTL’ by two-way ANOVA using Year and Genotype of 2DL-located QTL as variables (Interaction between Year and Genotype of 2DL-located QTL is considered in the model). *^b^* Kitahonami-2DL is a line bred by backcrossing with Kitahonami as the recurrent parent and ‘Sumai 3’ as the donor of the ‘Sumai 3’-derived allele at the 2DL-located QTL. Genotypes at the 2DL-located QTL are described in Table 1. The double asterisk and dagger indicate significant differences (P < 0.01 and P < 0.10, respectively). TGW, thousand grain weight.

**Table 3.** Genotypes of QTL of FHB-resistant breeding lines

Line	Genotype at the QTL		
	2DL	3BS	5AS
K-1932	c	a	b
K-1976	c	b	a
Sumai 3	a	a	a
Kitahonami	b	b	b

Genotypes at the QTL are described in Table 1.

**Table 4.** Effect of the ‘Asakaze’-derived allele on TGW in line selection and FHB resistance in FHB evaluation with sib lines

Line	Genotype of 2DL-located QTL*^a^*	TGW (g)	Severity of FHB*^c^*
W22261	c	43.9	5
W22265	c	45.4	13
W22266	c	36.9	9
W22263	Seg	44.3	7
W22264	Seg	40.0	5
W22262	b	42.4	23
W22267	b	37.6	55
**Average of lines**	**c**	**42.1**	**9**
	**Seg**	**42.2**	**6**
	**b**	**40.0**	**39**
	***P*-value*^b^***	**0.82**	**0.08^†^**

*^a^* Seg: alleles c and b are segregated in the line. *^b^* *P*-value is for the effect of ‘genotype of 2DL-located QTL’ by one-way ANOVA. *^c^* FHB resistance was evaluated separately from TGW evaluations. FHB severity is scored as Minimum 0 to Maximum 100. Genotypes at the 2DL-located QTL are described in Table 1. FHB, Fusarium head blight; TGW, thousand grain weight.

**Table 5.** Effect of the ‘Asakaze’-derived allele on agricultural traits in the yield trial and FHB resistance in the FHB evaluation with NILs

Genetic background*^a^*	Genotype of 2DL-located QTL	Days to heading	Days to maturity	Plant height (cm)	Yield (t/ha)	Yield percentage [c/b] (%)	TGW (g)	Difference in TGW [c – b] (g)	Severity of FHB*^d^*
W22308	c	255	300	94.2	10.26	95	41.9	+0.5	52
	b	253	300	94.9	10.85		41.4		65
W22309	c	255	300	89.6	9.02	105	42.0	–0.3	49
	b	254	300	88.6	8.61		42.3		58
**Average**	**c**	**255**	**300**	**91.9**	**9.64**	**99**	**42.0**	**+0.1**	**51**
	**b**	**254**	**300**	**91.7**	**9.73**		**41.9**		**61**
***P*-value*^b^***		**0.09^†^**	**n.d.*^c^***	**0.62**	**0.67**		**0.87**		0.01*

*^a^* Line name of genetic background of NILs. *^b^* *P*-value is for the effect of ‘Genotype of 2DL-located QTL’ by three-way ANOVA using genetic background, genotype of 2DL-located QTL and block (randomized block design) as variables (interaction between genetic background and 2DL-located QTL is considered in the model). *^c^* Not determined because all replications represent the same date. *^d^* FHB resistance was evaluated separately from the yield trial. FHB severity is scored as Minimum 0 to Maximum 100. Single asterisks and daggers indicate significant differences (P < 0.05, P < 0.10, respectively). Genotypes at the 2DL-located QTL are described in Table 1. FHB, Fusarium head blight; TGW, thousand grain weight.
