# Supplementary material for: Novel allele derived from a Japanese wheat cultivar ‘Asakazekomugi’ for Fusarium head blight resistance without yield penalty and thousand grain weight reduction
Source: Breed Sci. 2026 Feb 4;76(2):155–64. doi: 10.1270/jsbbs.24091 (PMC13494326; doi:10.1270/jsbbs.24091)
Supplement: Supplementary file 1 [file 76_155_s1.pdf]

doi: 10.1270/jsbbs.24091

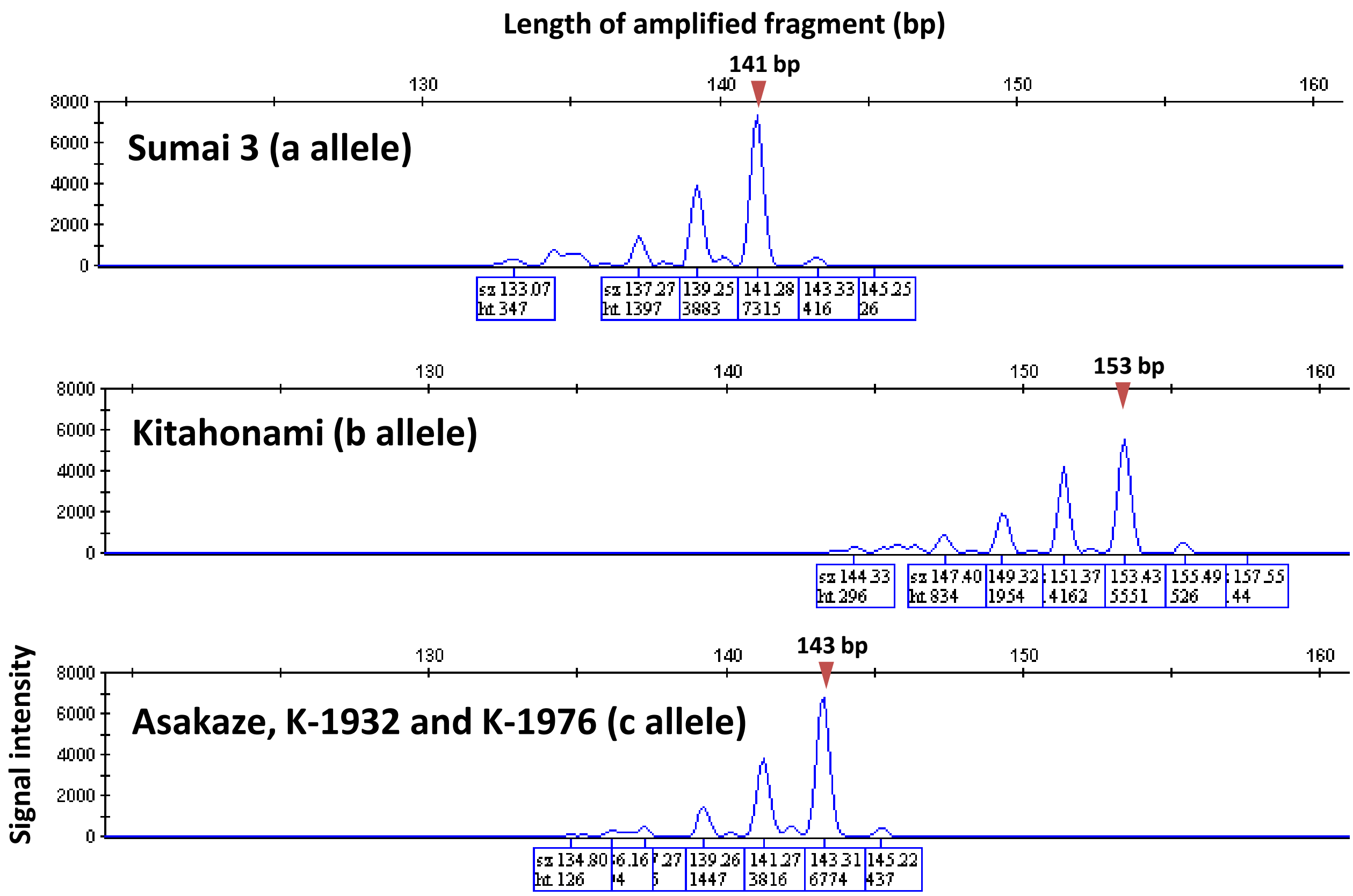

Supplemental Fig. 1 Length of the PCR-amplified fragment for *gwm539*

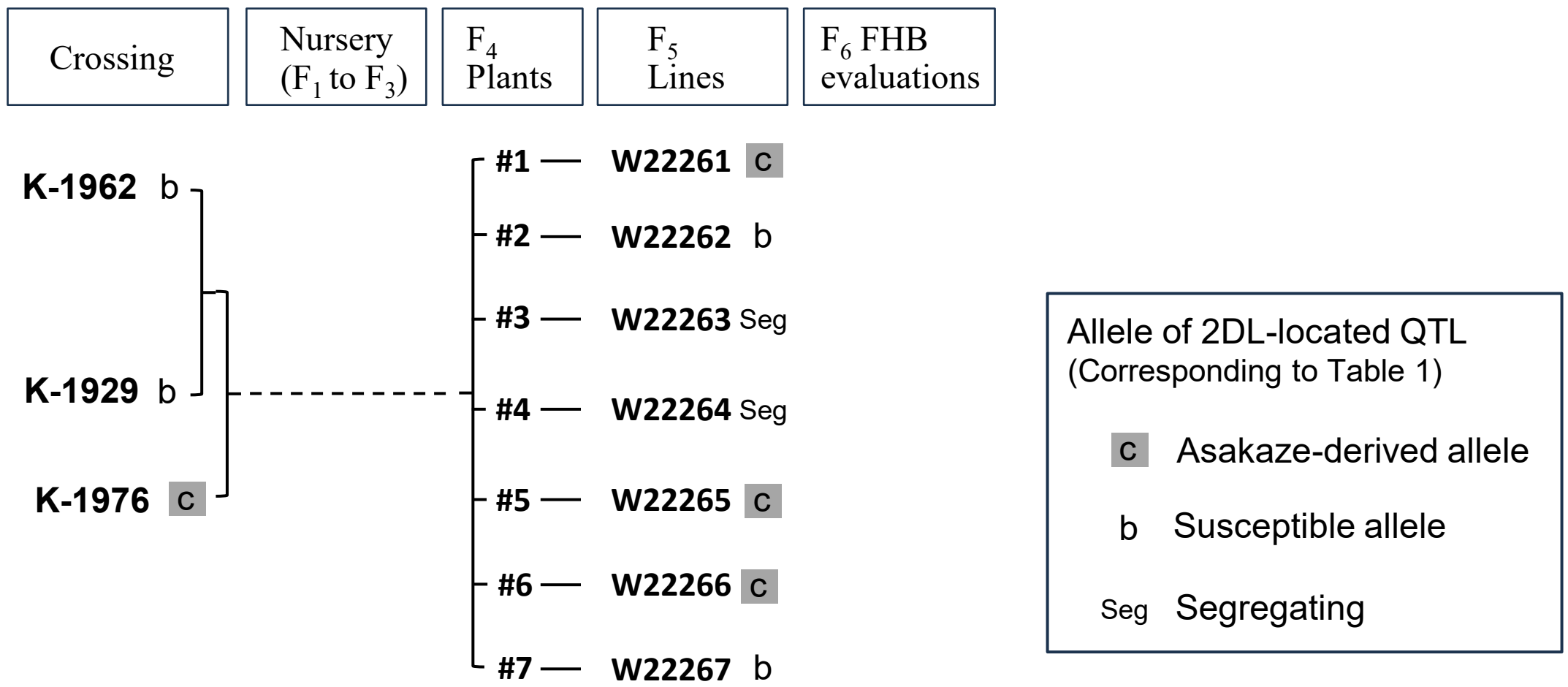

Supplemental Fig. 2 Outline of plant materials in Table 4

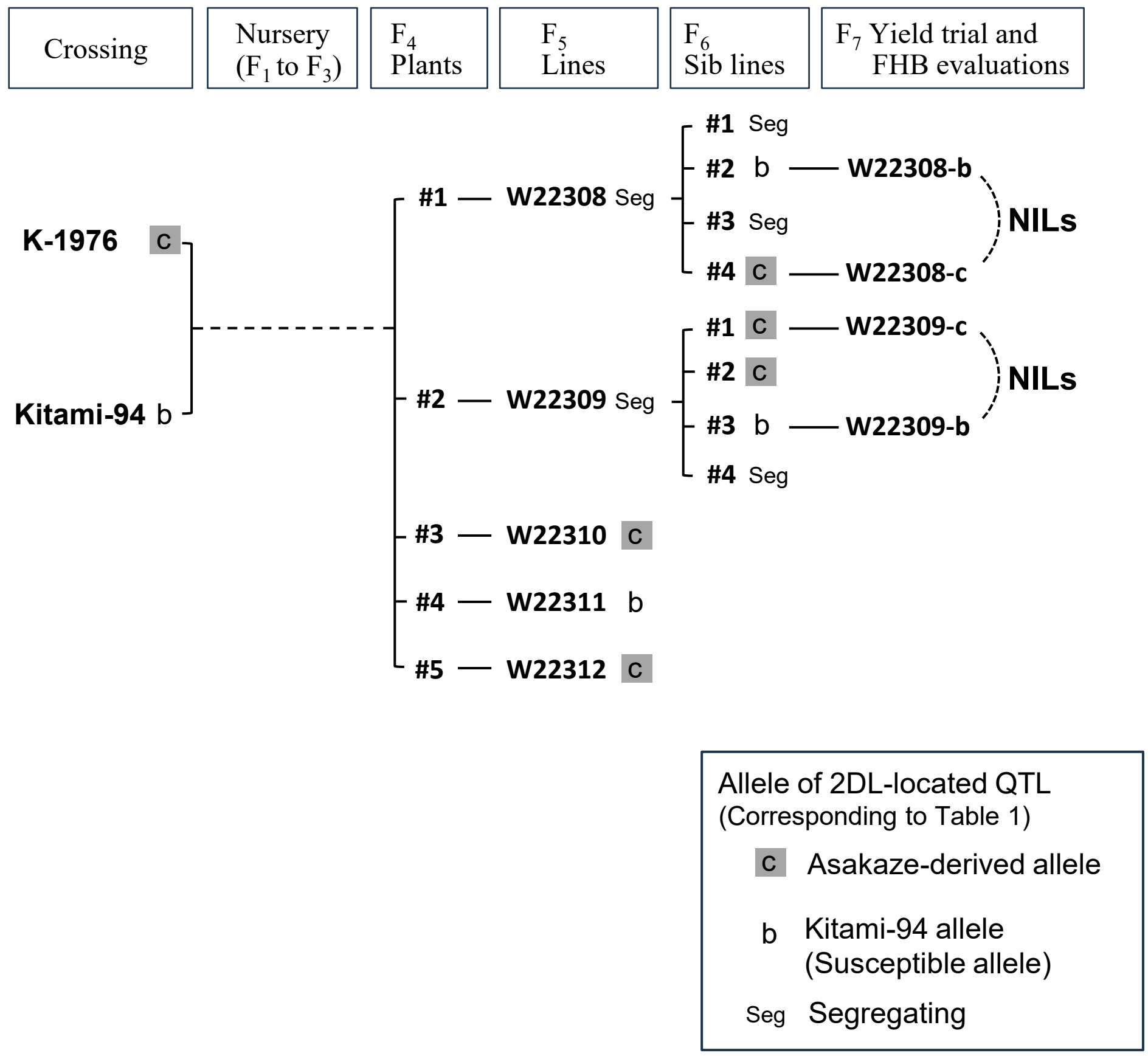

Supplemental Fig. 3 Outline of the two sets of NILs in Table 5
