# Supplementary material for: Novel allele derived from a Japanese wheat cultivar ‘Asakazekomugi’ for Fusarium head blight resistance without yield penalty and thousand grain weight reduction
Source: Breed Sci. 2026 Feb 4;76(2):155–64. doi: 10.1270/jsbbs.24091 (PMC13494326; doi:10.1270/jsbbs.24091)
Supplement: Supplementary file 2 [file 76_155_s2.pdf]

doi: 10.1270/jsbbs.24091

Supplemental Table 1 List of accession number for evaluated germplasm

Germplasm	Accession number of NARO Genebank
Asakazekomugi	JP21408
Ejimajinriki	JP21262
Fukuoka-18	JP21263
Hiyokukomugi	JP21305
Jyunreikomugi	JP21136
Nourin-59	JP21076
Nourin-61	JP21313
Saikai-95	JP21337
Saikai-104	JP21344
Saikai-165	JP46856
Saitama-29	JP20777
Shinchunaga	JP21063
Shirasagikomugi	JP21167
Shiroganekomugi	JP21414

Supplemental Table 2 Summary of experiments performed

	Type of experiments	Sowing year
Table 2	Yield trial	2014, 2015, 2016
Table 4	Line selection	2021
	FHB resistance (oats)	2022
Table 5	Yield trial	2023
	FHB resistance (spray)	2023
Fig. 1	FHB resistance (spray)	2014, 2015, 2016
Fig. 3	FHB resistance (oats)	2022

Supplemental Table 3 List of primes for DNA markers for genotyping FHB resistance QTLs

Locus	Type	Sequence
Xgwm539（2DL）	forward	ctgctctaagattcatgcaacc
	reverse	gaggcttgtgccctctgtag
Xgwm493（3B）	forward	ttcccataactaaaaccgcg
	reverse	ggaacatcatttctggactttg
Xgwm304（5A）	forward	aggaaacagaaatatcgcgg
	reverse	aggactgtggggaatgaatg

Supplemental Table 4 Overview of ANOVA design and results obtained

Name of Table	**Traits** /Source of Variation	degree of freedom	sum of squares	F value	*P* -value	Significance
**Table 2**	**Two-way ANOVA**					
	**Days to heading (days)**					
	Genotype of 2DL-located QTL (G)	1	0.04	0.20	0.67	
	Year (Y)	2	132.52	318.06	<0.01	**
	G×Y	2	0.10	0.23	0.80	
	**Days to Maturity (days)**					
	Genotype of 2DL-located QTL (G)	1	0.26	1.14	0.32	
	Year (Y)	2	57.61	125.69	<0.01	**
	G×Y	2	2.27	4.96	0.04	*
	**Plant height (cm)**					
	Genotype of 2DL-located QTL (G)	1	1.28	0.30	0.60	
	Year (Y)	2	351.80	41.48	<0.01	**
	G×Y	2	1.83	0.22	0.81	
	**Yield (t/ha)**					
	Genotype of 2DL-located QTL (G)	1	1.22	4.27	0.07	†
	Year (Y)	2	68.54	1.00	<0.01	**
	G×Y	2	0.08	0.14	0.88	
	**TGW (g)**					
	Genotype of 2DL-located QTL (G)	1	87.56	258.72	<0.01	**
	Year (Y)	2	98.81	145.99	<0.01	**
	G×Y	2	3.58	5.30	0.03	*
	**Thousand grains per square meter**					
	Genotype of 2DL-located QTL (G)	1	3.06	1.36	0.28	
	Year (Y)	2	356.27	78.94	<0.01	**
	G×Y	2	1.46	0.32	0.73	

Name of Table	Traits /Source of Variation	degree of freedom	sum of squares	F value	*P* -value	Significance
**Table 4**	**One-way ANOVA**					
	**TGW (g)**					
	Genotype of 2DL-located QTL	2	6.31	0.20	0.82	
	**FHB severity**					
	Genotype of 2DL-located QTL	2	1397.58	5.01	0.08	†
**Table 5**	**3-way ANOVA (Randomized block design)**					
	**Days to heading (days)**					
	Genotype of 2DL-located QTL (G)	1	0.02	0.22	0.67	
	Genetic background of NILs (B)	1	6.08	76.28	<0.01	**
	G×B	1	0.50	6.23	0.09	†
	Block of Yield trial	1	0.02	0.25	0.65	
	**Days to Heading (days)**					
	Genotype of 2DL-located QTL (G)	1	2.00	6.00	0.09	†
	Genetic background of NILs (B)	1	0.50	1.50	0.31	
	G×B	1	0.50	1.50	0.31	
	Block of Yield trial	1	2.00	6.00	0.09	†
	**Days to Maturity (days)**					
	Genotype of 2DL-located QTL (G)	1	0.00	-	-	
	Genetic background of NILs (B)	1	0.00	-	-	
	G×B	1	0.00	-	-	
	Block of Yield trial	1	2.00	-	-	
	**Plant height (cm)**					
	Genotype of 2DL-located QTL (G)	1	0.05	0.31	0.62	
	Genetic background of NILs (B)	1	59.41	405.03	<0.01	**
	G×B	1	1.45	9.85	0.05	*
	Block of Yield trial	1	30.42	207.41	<0.01	**
	**Yield (t/ha)**					
	Genotype of 2DL-located QTL (G)	1	0.02	0.22	0.67	
	Genetic background of NILs (B)	1	6.08	76.28	<0.01	**
	G×B	1	0.50	6.23	0.09	†
	Block of Yield trial	1	0.02	0.25	0.65	
	**TGW (g)**					
	Genotype of 2DL-located QTL (G)	1	0.02	0.03	0.87	
	Genetic background of NILs (B)	1	0.45	0.71	0.46	
	G×B	1	0.26	0.40	0.57	
	Block of Yield trial	1	0.46	0.71	0.46	
	**FHB**					
	Genotype of 2DL-located QTL (G)	1	231.13	32.44	0.01	**
	Genetic background of NILs (B)	1	45.13	6.33	0.09	†
	G×B	1	10.13	1.42	0.32	
	Block of FHB evaluation	1	703.13	98.68	<0.01	**
**Fig. 3**	**One-way ANOVA**					
	**FHB severity (KC-6153)**					
	Genotype of 2DL-located QTL	2	1332.17	5.33	0.01	**
	**FHB severity (KC-6055)**					
	Genotype of 2DL-located QTL	2	1049.18	3.62	0.04	*

Double asterisks, single asterisks, and daggers indicate significant differences ($P < 0.01$, $P < 0.05$, and $P < 0.10$, respectively).
